# Unveiling the *Trypanosoma cruzi* Nuclear Proteome

**DOI:** 10.1371/journal.pone.0138667

**Published:** 2015-09-18

**Authors:** Agenor de Castro Moreira dos Santos Júnior, Dário Eluan Kalume, Ricardo Camargo, Diana Paola Gómez-Mendoza, José Raimundo Correa, Sébastien Charneau, Marcelo Valle de Sousa, Beatriz Dolabela de Lima, Carlos André Ornelas Ricart

**Affiliations:** 1 Department of Cell Biology, Institute of Biology, University of Brasilia, Campus Darcy Ribeiro, Asa Norte, 70910–900, Brasília, Brazil; 2 Laboratório Interdisciplinar de Pesquisas Médicas, Instituto Oswaldo Cruz (IOC), Fundação Oswaldo Cruz (FIOCRUZ), 21040–360 Manguinhos, Rio de Janeiro, RJ, Brasil; University of Sao Paulo, BRAZIL

## Abstract

Replication of *Trypanosoma cruzi*, the etiological agent of Chagas disease, displays peculiar features, such as absence of chromosome condensation and closed mitosis. Although previous proteome and subproteome analyses of *T*. *cruzi* have been carried out, the nuclear subproteome of this protozoan has not been described. Here, we report, for the first time to the best of our knowledge, the isolation and proteome analysis of *T*. *cruzi* nuclear fraction. For that, *T*. *cruzi* epimastigote cells were lysed and subjected to cell fractionation using two steps of sucrose density gradient centrifugation. The purity of the nuclear fraction was confirmed by phase contrast and fluorescence microscopy. Liquid chromatography coupled to tandem mass spectrometry (LC-MS/MS) allowed the identification of 864 proteins. Among those, 272 proteins were annotated as putative uncharacterized, and 275 had not been previously reported on global *T*. *cruzi* proteome analysis. Additionally, to support our enrichment method, bioinformatics analysis in DAVID was carried out. It grouped the nuclear proteins in 65 gene clusters, wherein the clusters with the highest enrichment scores harbor members with chromatin organization and DNA binding functions.

## Introduction

*Trypanosoma cruzi*, a protozoan that belongs to the Trypanosomatidae family, causes Chagas disease, the American trypanosomiasis, which affects approximately 7–8 million people worldwide, especially in Latin America [1]. Chagas disease is mainly transmitted by triatomine bugs, but it can also be disseminated through blood transfusion, organ transplant, vertical transmission from mother to offspring, ingestion of contaminated food and laboratory accidents [1].

*T*. *cruzi* possesses a digenetic life cycle, differentiating into four main life forms: epimastigote and metacyclic trypomastigote, which are found in the insect vectors, and trypomastigote and amastigote, which occur in mammalian hosts. In epimastigote, the life form studied here, the kinetoplast (a mitochondrial complex array of DNA fibrils) is localized anterior to the nucleus [2].

*T*. *cruzi* and other trypanosomatids have peculiar cell nucleus features when compared to other eukaryotes, such as animals. Some examples of these features are the absence of chromosome condensation and distribution in mitosis [3], closed mitosis (i.e. the nuclear envelope remains intact) [4] and replication of the DNA in the periphery of the nucleus [5]. In spite of the fact that these features have been described earlier, little is known about cell replication processes, as well as the proteins involved in these mechanisms.

*T*. *cruzi* presents polycistronic transcription and its gene expression is post-transcriptionally regulated, leading to a poor correlation between mRNA and protein levels [6]. Therefore, proteomics can be considered a reliable tool for the study of gene product expression in this parasite. Previous proteomic studies have been carried out aiming the understanding of *T*. *cruzi* biology, including global proteomic analysis [7, 8], proteomic changes during cell differentiation [9, 10] and analysis of subproteomes such as secretome [11], cell surface [12, 13], and organelles [14]. Here, we present for the first time, to the best of our knowledge, the isolation and proteome analysis of *T*. *cruzi* nuclear fraction.

## Material and Methods

Fetal bovine serum (FBS) was purchased from Gibco, South America, and modified trypsin was acquired from Promega (Madison, WI, USA). All others reagents were purchased from Sigma-Aldrich (St. Louis, MO, USA), unless stated otherwise.

### Parasite culture

*T*. *cruzi* (CL Brener strain) epimastigotes were cultivated at 28°C in Liver Infusion Tryptose medium (LIT) [15] supplemented with 10% (v/v) Fetal Bovine Serum (FBS), 100 μg/ml ampicillin and 100 μg/ml streptomycin and 2 mg/mL hemin until the cells reached the logarithmic phase.

### Isolation of *T*. *cruzi* nuclear fraction

The methodology developed for the production of *T*. *cruzi* nuclear fraction was based on protocols previously described [16, 17]. For CL-Brener cell counting, a Neubauer chamber was used and approximately 1.0 x 10^10^ epimastigote cells were used for each nuclear isolation procedure. The following procedure was carried out independently for two biological replicates. All steps were performed at 4°C. Cells were centrifuged at 5,000 *g* for 10 min and then washed three times in Phosphate Buffered Saline (PBS). Pellet was resuspended in eight volumes of hypotonic buffer TENM2 (10 mM Tris-HCl pH 7.4, 10 mM NaCl, 1 mM MgCl_2_, 1 mM MnCl_2_, 5 mM β-mercaptoethanol) and cell turgidity was confirmed by optical microscopy. Nonidet P40 0.5% (v/v) final concentration and protease inhibitors (Complete Mini, Roche, Meylan, France) were added to the cells, which were then lysed using a Dounce homogenizer. Cell lysis was followed by optical microscopy. Osmolarity of the lysate was reestablished by adding 12.5% (v/v) of 2 M sucrose (final sucrose concentration 0.25 M). It was then transferred to a conical centrifuge tube containing 5 ml of 0.58 M sucrose in TENM2 and centrifuged at 2,000 *g* for 10 min. The top layer containing the cytosol was carefully removed. The pellet, which contained a high density organelle fraction, was resuspended with 16 ml of 1.9 M sucrose in TENM2. The suspension was loaded on a centrifuge tube (SW28 Beckman) containing a discontinuous step gradient (from the bottom, 8 ml of 2.30 M sucrose, 8 ml of 2.10 M sucrose and 8 ml of 2.01 M sucrose; all steps in TENM2 with protease inhibitors). The tube was centrifuged at 141,000 *g* in a SW28 rotor for 3 h at 4°C. Fractions were immediately unloaded from the top, stained with the blue- fluorescent 4',6-diamidino-2-phenylindole (DAPI), examined by phase contrast and fluorescence microscopy in a TCP SP5 confocal microscope (Leica Microsystems, Wetzlar, Germany) and stored at -80°C.

### SDS-PAGE and identification of protein bands by 1D-LC-MS/MS

Equal amounts of each sample (cytosol, kinetoplast and nucleus), corresponding to 30 μg of protein per lane, were separated on 13% T SDS-PAGE gels using a SE 600 electrophoresis system (Hoefer, Inc, San Francisco, CA, USA). Electrophoresis was performed at 15 mA at 18°C. Next, gels were stained with Coomasie Blue G 250 (CBB-G 250) and digitalized in Image Scanner (GE Healthcare, Little Chalfont, United Kingdom) using a Power Look Software (GE Healthcare).

Protein bands of interest were excised from the gel and washed twice with 50% (v/v) acetonitrile, followed by reduction with 10 mM dithiotreitol (DTT) in 10 mM NH_4_HCO_3_ pH 8.0 at 56°C for 1 hour. Cystein carbamidomethylation of each sample was performed with 55 mM iodoacetamide (IAA) for 1 h at room temperature in the dark. Gel slices were then rinsed three times with 10 mM NH_4_HCO_3_ pH 8.0 and dehydrated with 100% (v/v) ACN. After vacuum drying, the gels were rehydrated with digestion buffer (12.5 ng/μL modified trypsin in 25 mM NH_4_HCO_3_ pH 8.0, 5 mM CaCl_2_) and the proteolytic digestion was performed overnight at 37°C. The peptides were recovered from gelby three cycles of 10 min incubation with ACN: H_2_O:TFA (66:33:0.1 v/v/v) and 10 min sonication. Extracts were dried in a vacuum centrifuge, resuspended in loading solution (3% ACN:97% H_2_O: 0.1% formic acid) and injected onto a Q-TOF micro mass spectrometer (Waters, Manchester, UK).

Following the injection of the sample at a 10 uL/min flow rate for 15 min, the peptides were captured onto the reversed-phase (RP) trap column (180 μm × 20 mm) packed with 5 μm Symmetry C18 material (Waters, Milford, MA, USA). As the linear gradient started, the eluted peptides were separated using a nanoACQUITY BEH130 C18 1.7 um, 75um x 150mm RP analytical column (Waters, Milford, MA, USA). The mobile phases used for the RP chromatography were 0.1% formic acid in water (mobile phase A) and 0.1% formic acid in acetonitrile (mobile phase B). The total chromatography time was set to 70 min and the initial condition was 97% A and 3% B. Gradient increased linearly from 3 to 10% B in 3.67 min, then to 40% B from 3.67 to 50.79 min, and up to 85% B from 50.79 to 54.79 min. The gradient was maintained in 85% B from 54.79 to 58.79 min, followed by decreasing to 3% B from 58.79 to 60.79 min, and finally re-equilibration of the column to the initial condition till 70 min at a flow rate of 500 nL/min.

For the LC-MS/MS analysis, DDA acquisition mode was performed. Capillary and cone voltage was set at 3500 and 30 V, respectively. For the MS survey, the scan time was set to 1 s with inter-scan delay at 0.1 s and the range was from 400 to 2000 Da. The MS survey switched to MS/MS acquisition when the intensity of the precursor ion reached above 40 counts per second. For the MS/MS, the maximum number of selected precursor ions for fragmentation was three ions of charge 2, 3 or 4 detected from a single MS survey scan. MS/MS was obtained at a scan rate of 2 s and inter-scan delay at 0.1 s, acquired over the range from 50 to 2000 Da. MS/MS acquisition returned to MS survey when the peak intensity fell below 4 counts per second or after 4.2 seconds have elapsed. Collision energy was ramped according to the *m/z* and charge state of the precursor ion, controlled by the charge state recognition files. The TOF analyzer was calibrated with phosphoric acid (Sigma-Aldrich, St. Louis, MO, USA) (GFP, 100 fmol/uL in 50:50:1, metanol:H_2_O:acetic acid) as the reference solution from 50 to 2000 m/z. Singly-charged cluster ion *m/z* 585 was used as lock mass correction, and lock spray reference scan frequency was set to every 15 s during the acquisition at a scan of 1 second.

### Extraction and trypsin digestion of *T*. *cruzi* nuclear proteins for 2D-LC-MS/MS

*T*. *cruzi* nuclear fraction replicates were resuspended in 7 M urea/2 M thiourea at 4°C and homogenized using a vortex mixer (5 times 20 s with intervals of 5 min in ice bath). The nuclei lysis was monitored by optical microscopy. Each lysate was centrifuged for 10 min at 15,000 *g*. The protein concentration of the supernatant was quantified using a *Qubit*® Protein Assay Kit (Life Technologies, Eugene, Oregon, USA) according to manufacturer's instructions, and the extracts were stored at– 80°C until use.

Before trypsin digestion, a volume of 4 μL of 1 M NH_4_HCO_3_ pH 8.0 was added to 36 μL of nucleus extract (80 μg of protein) followed by the addition of 4.4 μL of 100 mM DTT and incubation at 30°C for 1 h. For cystein carbamidomethylation, 5 μL of 400 mM IAA was added to the sample, which was incubated at room temperature for 30 min in the dark. Subsequently, 250 μL of ultrapure H_2_O were added to lower urea and thiourea concentration. Modified trypsin was then added to reach a final trypsin:substrate ratio of 1:50. Finally, samples were incubated overnight at 39°C. The resulting tryptic peptides were acidified with 0.1% TFA and desalted using C18 Ultra-Micro Spin columns (Harvard Apparatus, Holliston, MA, USA). The resulting samples were dried in a vacuum centrifuge.

### 2D-LC-MS/MS

Prior to 2D-LC-MS/MS, the tryptic peptide samples from two biological replicates were resuspended in 40 μL and 25 μL of anion exchange loading buffer (5 mM ammonium formate, 5% ACN pH 3.2), respectively, to a final peptide concentration of 2 μg/uL. The peptide mixtures were analyzed in nanoLC/MS^E^ acquisition mode using a SYNAPT^TM^ G1 HDMS™ System (Waters, Manchester, UK) and mass spectra were acquired in positive ion TOF V-mode. TOF analyzer was calibrated with the MS/MS fragment ions of [Glu1]-fibrinopeptide B (Sigma-Aldrich, St. Louis, MO, USA) (GFP, 100 fmol/uL in 50:50:1, metanol:H_2_O:acetic acid) from 50 to 2000 m/z. GFP double-charged precursor ion at *m/z* 785.8426 was used as lock mass correction for accurate MS post acquisition measurements. During each LC-MS/MS run, the reference sprayer (GFP) was injected once every 30 s and acquired for 1 s. The mass spectra were acquired alternating low and high collision energy during 0.8 s in each mode and 0.02 s interscan delay time. In the low-energy MS mode data were collected at constant collision energy of 4 eV, whereas in the high-energy MS mode the collision energy ramped from 15 to 55 eV. Capillary and cone voltage was set at 3000 and 35 V, respectively. MS survey scan range was from 300 to 2000 Da and MS/MS was acquired over the range from 50 to 2000 Da.

Digested peptide mixtures were loaded onto a Waters nanoACQUITY UPLC system coupled to the mass spectrometer. Chromatography setup was based on the method by [18], consisting of an online two-dimensional (2D) nano-scale liquid chromatography. The first dimension chromatography included a pre-packed 180 μm × 20 mm strong cation-exchange (SCX) column (nanoACQUITY UPLC SCX TRAP Column) (Waters, Milford, MA, USA) in conjunction with a trap column (180 μm × 20 mm) packed with 5 μm Symmetry C18 material (Waters, Milford, MA, USA). For the second dimension, a nanoACQUITY BEH130 C18 1.7 um, 75um x 150mm reversed-phase (RP) analytical column (Waters, Milford, MA, USA) was used. An isocratic auxiliary pump (or ASM) allowed the SCX column to be equilibrated with loading buffer and performed the step gradient runs by loading 9 uL of “salt plugs” onto the SCX columns. The “plug” solutions (fractions) contained both ammonium formate and acetonitrile in varied concentration. The eight fractions were prepared from a 1M ammonium formate stock solution, pH 3.2 and acetonitrile according to the following: three fractions (50, 100 and 150 mM ammonium formate) containing 5% acetonitrile, four 200 mM ammonium formate buffers with 5, 10, 20 and 30% acetonitrile, and a FLUSH solution (350 mM ammonium formate, 50% acetonitrile). Following the injection of the fractions at a 5 uL/min flow rate for 10 min, the released peptides from the SCX column were captured into the C18 trap column, while the eluted ones were separated on the reversed-phase analytical column using a linear gradient of 0.1% formic acid in water (mobile phase A) and 0.1% formic acid in acetonitrile (mobile phase B) for 80 min performed by the binary pump (BSM). Chromatography initial condition was set at 95% A and 5% B, and the gradient increased linearly from 5 to 50% B in 56.8 min, then to 85% B from 56.80 to 59.80 min, and maintain it in 85% B from 59.80 to 62.80, followed by decreasing to 5% B from 62.80 to 65.80 min, and finally re-equilibration of the column (95% A and 5% B) from 65.80 to 80 min at a flow rate of 300 nL/min.

### Data Analyses

For the MS^E^ acquisition mode, mass spectra were processed using the Protein LynxGlobal Server version 2.5.1 (PLGS) (Waters, Manchester, UK), and proteins were identified using the embedded Identity^E^ algorithm. MS/MS spectra were searched against the *Trypanosoma cruzi* CL Brener database containing reverse sequences (http://www.uniprot.org; release oct_2014). For protein search, the parameters used were carbamidomethylation of cysteine as fixed modification and the variable modifications were acetylation of the N-terminal, deamidation of asparagines/glutamine, pyro-glutamine, pyro-glutamate and oxidation of methionine. One trypsin missed cleavage site was allowed and the precursor and fragment ion tolerances were 10 and 20 ppm, respectively. Protein identification criteria included the detection of at least three fragment ions per peptide and seven per protein, and at least one peptide per protein hit. False-positive discovery rate was set at 1%. Components were typically clustered against a theoretical peptide ion mass database using a mass precision of 10 ppm and a time tolerance of 0.25 min. Elevated energy ions were aligned with low-energy precursor peptide ions using an approximate precision of 0.05 min. Bioinformatics analyses were performed using Blast2GO software (http://www.blast2go.com/b2ghome) and DAVID (Database for Annotation, Visualization and Integrated Discovery) Bioinformatics Resources v6.7 (http://david.abcc.ncifcrf.gov/)[19].

## Results and Discussion

The procedure used here to isolate *T*. *cruzi* nuclear fraction was adapted from two previously described methods. One of them permitted the isolation of *T*. *cruzi* high density organelles [16], while the other was developed for the purification of *T*. *brucei* nucleus in order to produce samples suitable for proteomic analysis [17].

**Fig 1:**
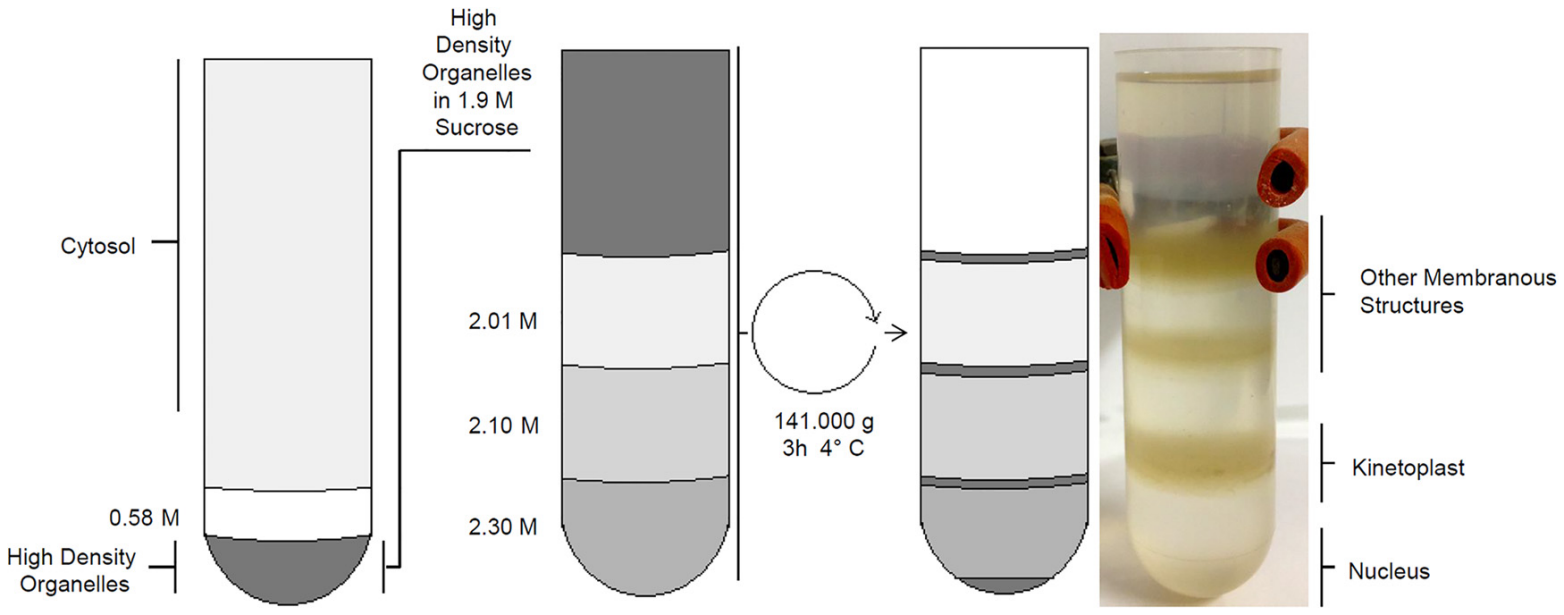
*T*. *cruzi* epimastigote subcellular fractionation. Sucrose gradient steps and isolated fractions are indicated.

Following lysis of epimastigote life forms, a fraction containing *T*. *cruzi* epimastigote high density organelles was pelleted by centrifugation on 0.58 M sucrose. This fraction was then subjected to sucrose density gradient ultracentrifugation, which resulted in a pellet and three interfaces as shown in Fig 1.

**Fig 2:**
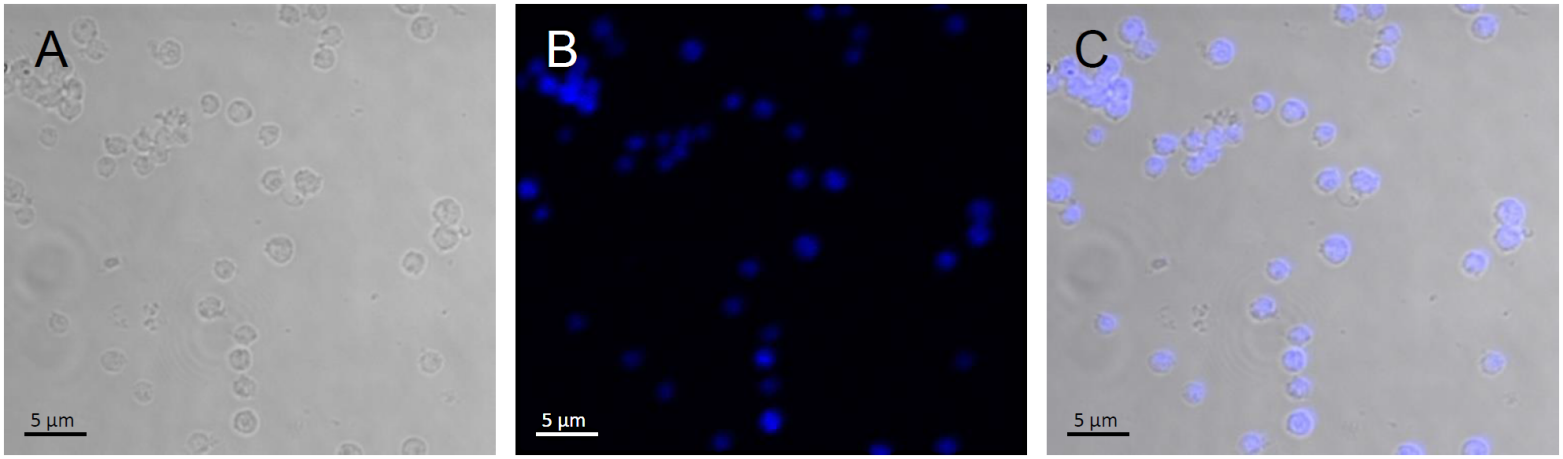
Phase contrast and fluorescence microscopy analysis of *T*. *cruzi* nuclear fraction. A—Phase contrast, B—DAPI staining, C—Merge. The round structures correspond to nuclei.

Since *T*. *brucei* and *T*. *cruzi* display several morphological differences, the protocol developed by [17] did not provide identical results when applied to *T*. *cruzi*. For example, after ultracentrifugation, *T*. *brucei* nuclear fraction was concentrated in the interface between 2.30 M and 2.10 M sucrose[17], while *T*. *cruzi* nuclear fraction was found in the pellet (Fig 1). Analysis of this fraction by phase contrast microscopy showed rounded structures with diameter of 2.5 μm, presenting more refringent spots inside, compatible with nucleolus. DAPI® labeling and fluorescence microscopy demonstrated the presence of DNA inside those structures (Fig 2). Phase microscopy also showed the absence of kinetoplasts, networks of circular DNA that occur inside mitochondria, in *T*. *cruzi* nuclear fraction. Kinetoplasts, which are significantly smaller than the nuclei [2], were recovered in the interface between 2.1 M and 2.01 M sucrose layers, while the interface above sucrose 2.01 M contained other membranous structures (data not shown).

**Fig 3:**
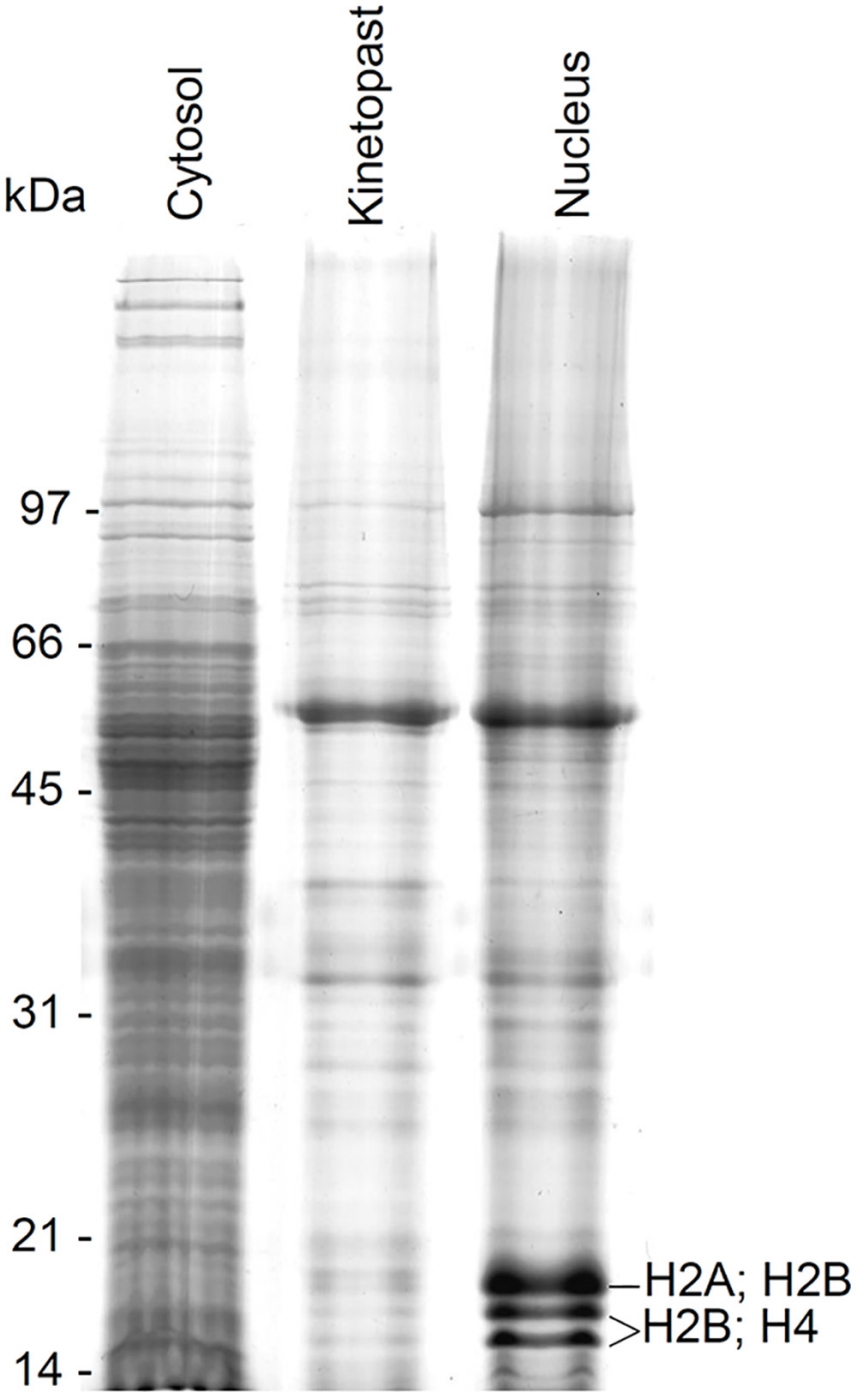
SDS-PAGE of *T*. *cruzi* subcellular fractions. Epimastigote cells were lysed and subjected to cell fractionation as described in Material and Methods. Cytosolic, kinetoplastic and nuclear samples (30μg/lane) were subjected to 13% T SDS-PAGE. The gel was stained with Coomassie Brilliant Blue.

The protein profiles of nuclear, cytoplasmic and kinetoplast fractions were compared by SDS-PAGE (Fig 3). The nuclear fraction profile displayed three bands between 14 and 21 kDa which were less intensely stained in the other samples. Similar protein band profiles were previously reported in *T*. *brucei* nuclear fraction and were assumed as histones [17], based solely on the molecular mass range. In order to identify those protein bands, they were subjected to mass spectrometric analysis which revealed that those are the typical nuclear histone proteins H2, H3 and H4 (Fig 3). This finding associated with microscopy analysis (Fig 2) ensured the high efficiency of the cell fractionation process used in this work.

Proteome analysis of the *T*. *cruzi* nuclear fraction by 2D-LC-MS/MS provided the identification of 864 proteins (S1 Table) of 12875 peptides (S2 Table). The first large scale *T*. *cruzi* proteome analysis [8] described 1,573 epimastigote proteins and a total of 2,784 proteins from the four parasite life-stages. Here, the identification of 275 proteins that were not reported by in that work [8] was achieved (S3 Table), demonstrating that isolation of *T*. *cruzi* nuclear fraction permitted the enrichment of proteins which were underrepresented in global proteomic studies [20]. Therefore, proteins involved in vital nuclear biological processes, i.e. centrins, histones, nucleolar proteins, nucleosome assembly proteins, ribosome proteins, transcription factors and structural nuclear proteins were identified. Examples of peptide mass spectra (MS/MS) of centrin and histones can be seen in S1 File.

Centrins are calcium-binding proteins that were shown to play important roles in cell replication, centrosome stability and nuclear architecture in *Dictyostelium discoideum* [21]. They have been shown to be involved in the growth and division of Trypanosomatids. For instance, the expression of a mutant form of centrin reduced the growth of the *Leishmania donovani*, [22] while in *T*. *brucei* the depletion of TbCentrin4 resulted in the dysregulation of nuclear and cell division [23].

Several proteins related to RNA processing were identified such as poly A binding protein, RNA helicases, elongation factors and eukaryotic translation initiation factors. Fifty-three ribosomal proteins were identified in the nuclear fraction, and 25 of them were identified as components of the 40S ribosomal subunit and other 28 as components of the 60S subunit. Presence of ribosomal proteins in the nuclear fraction was expected since ribosome biosynthesis is a process that takes place mainly in the nucleoli and nucleoplasm [24].

Some membrane proteins involved in host-parasite interactions, such as mucins, trans-sialidases and kinetoplastid membrane proteins (KMP) were also identified. In *T*. *cruzi* mucins are the main acceptors of sialic acid via a trans-sialidase reaction. However, different mucins are produced depending on the parasite life stage, suggesting that they might have other functions [25]. Kinetoplastid membrane proteins (KMP) are described as flagellar proteins conserved among kinetoplastids. Previous work showed that gene silencing of *T*. *brucei* KMP-11 by RNA interference inhibited basal body segregation and cytokinesis, resulting in parasites with multiple nuclei of various sizes, which indicates a continuous and defective nuclear division while cell division was blocked.[26]. Moreover, those protein families were efficiently used to produce a Leishmania vaccine [27, 28], and the identification of KMPs in *T*. *cruzi* nuclear subproteome reinforce that their role are not limited to parasite-host interactions.

The data also revealed that *T*.*cruzi* nuclear subproteome is composed of several members of heat shock proteins and the retrotransposon hot spot (RHS). Heat shock proteins (HSP) are involved in homeostasis in the course of stress treatment [29] and have been identified before in most *Trypanosome* proteome and subproteome analyses [7–9, 29, 30], including nuclei [31]. HSP have also been considered as potential targets to design new drugs against African trypanosomiasis [32]. RHS proteins, on the other hand, were described as one of the largest gene family in *T*. *cruzi* genome [33]. Western blot analysis in *T*. *brucei* has shown that RHS proteins are constitutively expressed and occur mainly in the nucleus [34]

Some enzymes involved in carbohydrate and lipid metabolism were also identified in the present work. Those proteins are normally found in cell compartments other than nucleus. However, previous reports have showed that a large number of proteins are present in more than one cellular compartment. For example, in mouse liver cells it was demonstrated that 39% of all organellar proteins present in multiple cellular locations [35]. Additionally, it is known that there is a close functional relationship between *T*. *cruzi* organelles, which is frequently observed in subcellular fractionation studies [36].

The list of proteins identified in the present work was subjected to bioinformatics analysis aiming at assigning to them theoretical molecular functions and predicted cell localization. Gene Ontology (GO) analysis using Blast2GO software provided a limited amount of information since only 238 proteins (less than 30% of total) were assigned to a cell compartment. Analysis was also carried out using DAVID bioinformatic tools to determine gene functional classification as well as enrichment scores to specific clusters.

Thus, DAVID analysis (functional annotation clustering) provided 65 gene clusters (S4 Table), wherein the two clusters with the highest enrichment scores (28.9 and 22.9) harbor gene members that have nucleosome assembly and organization and DNA/RNA binding as enriched functional annotations. The next enriched cluster (transmembrane transporter activity) presented an enrichment score significantly lower than the first two (Table 1).

**Table 1:** Top 2 most representative clusters of nuclear proteome by DAVID bioinformatics resources.

Annotation Cluster 1 Enrichment Score: 28.91298942880829		Count	P-Value	Benjamini
GOTERM_BP_FAT	chromatin assembly	41	3.5E-31	8.6E-29
GOTERM_BP_FAT	protein-DNA complex assembly	41	3.5E-31	8.6E-29
GOTERM_BP_FAT	nucleosome organization	41	3.5E-31	8.6E-29
GOTERM_BP_FAT	DNA packaging	41	3.5E-31	8.6E-29
GOTERM_BP_FAT	nucleosome assembly	41	3.5E-31	8.6E-29
GOTERM_BP_FAT	chromatin assembly or disassembly	41	2.0E-29	2.4E-27
GOTERM_BP_FAT	chromatin organization	41	8.0E-27	6.5E-25
GOTERM_BP_FAT	chromosome organization	41	6.3E-25	3.9E-23
Annotation Cluster 2 Enrichment Score: 22.966985589113087		Count	P-Value	Benjamini
INTERPRO	Histone core	38	1.3E-35	3.2E-33
INTERPRO	Histone-fold	38	7.2E-34	1.2E-31
SP_PIR_KEYWORDS	chromosomal protein	38	2.4E-30	2.0E-28
GOTERM_CC_FAT	Chromatin	38	6.2E-21	5.7E-19
GOTERM_CC_FAT	Nucleosome	38	6.2E-21	5.7E-19
GOTERM_CC_FAT	protein-DNA complex	38	1.2E-19	5.5E-18
GOTERM_CC_FAT	chromosomal part	38	4.1E-17	1.2E-15
GOTERM_CC_FAT	Chromosome	38	4.5E-11	6.8E-10

A significant proportion of identified *T*. *cruzi* nuclear fraction proteins, 272 proteins corresponding to 31%, were annotated as putative uncharacterized *T*. *cruzi* proteins with unknown functions. This is in agreement with previous *T*. *cruzi* proteome analysis [10]. Possibly, these proteins are involved in nucleus-associated processes such as gene regulation, DNA replication and cell division molecules. Therefore, they should be studied as potential targets for developing new drugs.

Bioinformatics analysis of the putative uncharacterized *T*. *cruzi* nuclear proteins using DAVID classified them into 8 functional annotation clusters. The cluster bearing the highest enrichment score (5.8) grouped ALBA RNA/DNA binding proteins (Table 2). ALBA (Acetylation lowers binding affinity) proteins were recently described in the protozoan *Plasmodium falciparum*, where they perform a role in non-coding RNA and the regulation of antigenic variation [37].

**Table 2:** The cluster of most enriched uncharacterized putative proteins by DAVID bioinformatics resources.

Annotation Cluster 1 Enrichment Score: 6.663417037371656		Count	P-Value	Benjamini
INTERPRO	Alba, DNA/RNA-binding protein	6	5.4E-10	4.3E-8
PIR_SUPERFAMILY	PIRSF030333:DNA/RNA-binding protein related to Alba	4	1.0E-6	1.0E-5
PIR_SUPERFAMILY	PIRSF030333:UCP030333_Alba	4	1.0E-6	1.0E-5
INTERPRO	Uncharacterised conserved protein UCP030333, DNA/RNA-binding Alba-related	4	3.9E-6	1.5E-4

In *T*. *brucei*, cytoplasmic ALBA proteins that regulate translation initiation were identified [38]. However, the members of this protein family were first described as DNA-binding proteins in Archaea [39] and were shown to be associated with nuclear RNase MRP/P in yeast and mammals [40]. Existence of nuclear Alba proteins in *T*. *cruzi* could indicate intriguing differences between gene expression machinery in both trypanosomatids, which should be investigated in the future. Nonetheless, bioinformatic methods demonstrated the quality of our nuclear fraction and provided strong evidences that many putative uncharacterized proteins could participate of *T*. *cruzi* gene regulation processes.

Other putative uncharacterized proteins were clustered in functional groups such as cell cycle regulation and transcriptional control. Proteins with WD repeat were found among uncharacterized proteins, and one of them was described in *T*. *brucei* as having WD repeat elements homologue of receptor for activated C kinase 1 (RACK1) called TRACK. This protein is distributed predominantly in a perinuclear region and cytoplasm, but not along the endoplasmic reticulum and mitochondrion, and its function is to control the final stages of mitosis [41].

## Conclusions

The analysis of *T*. *cruzi* epimastigote nuclear subproteome was only possible because a specific cell fractionation procedure was optimized. The use of a purified nuclear fraction certainly enhanced the concentration of nuclear proteins that would be underrepresented in whole cell proteome analysis. The procedure also provided other subcellular fractions (e.g. kinetoplasts) that could be used in further subproteomic studies such as quantitative experiments and the identification of post-translational modifications in specific subcellular fractions.

Overall, more than 800 proteins, including putative uncharacterized proteins, were identified and assigned to *T*. *cruzi* nucleus. The approach used here may also be applied to study the nuclear fraction of other *T*. *cruzi* life stages, such as amastigote and trypomastigote. Finally, we believe that the characterization of *T*. *cruzi* nuclear subproteome may contribute to delineate a complete picture of the parasite proteome and to understand central processes that occur during its life cycle, including replication and differentiation.

## Supporting Information

S1 File Examples of peptide mass spectra (MS/MS): Centrin and Histones. (PDF)

S1 Table Proteins that compose the nuclear proteome of *T*. *cruzi* epimastigote form. (XLSX)

S2 Table LC-MS/MS peptide analysis data of T. cruzi nuclear fraction. (XLSX)

S3 Table Proteins of nuclear proteome not found in the large scale *T*. *cruzi* proteome analysis. (XLSX)

S4 Table Clusters assigned by DAVID bioinformatics resources. (XLSX)
