# Supplementary material for: Unveiling the Trypanosoma cruzi Nuclear Proteome
Source: PLoS One. 2015 Sep 18;10(9):e0138667. doi: 10.1371/journal.pone.0138667 (PMC4575177; doi:10.1371/journal.pone.0138667)
Supplement: S1 File — (PDF) [file pone.0138667.s001.pdf]

S1 File. Examples of peptide mass spectra (MS/MS): Centrin and Histones

Protein entry	Protein description	# Peptides	Peptide sequence	Peptide score
Q4DQ49	Centrin_ putative Trypanosoma cruzi (strain CL Brener) Tc00.1047053508323.60 4 1	8	AFQLFDLDKK	7,6496
			GLGFGDLPR	7,3367
			GLGFGDLPRDEVER	6,7946
			AFQLFDLDK	7,0327
			EAFNLFDADGSGAIDAEEM ALAMK	6,7973
			TMSTDSNGLIGYGEFER	6,9267
			AALTDEQIR	7,6967
			TMSTDSNGLIGYGEFER	6,5735

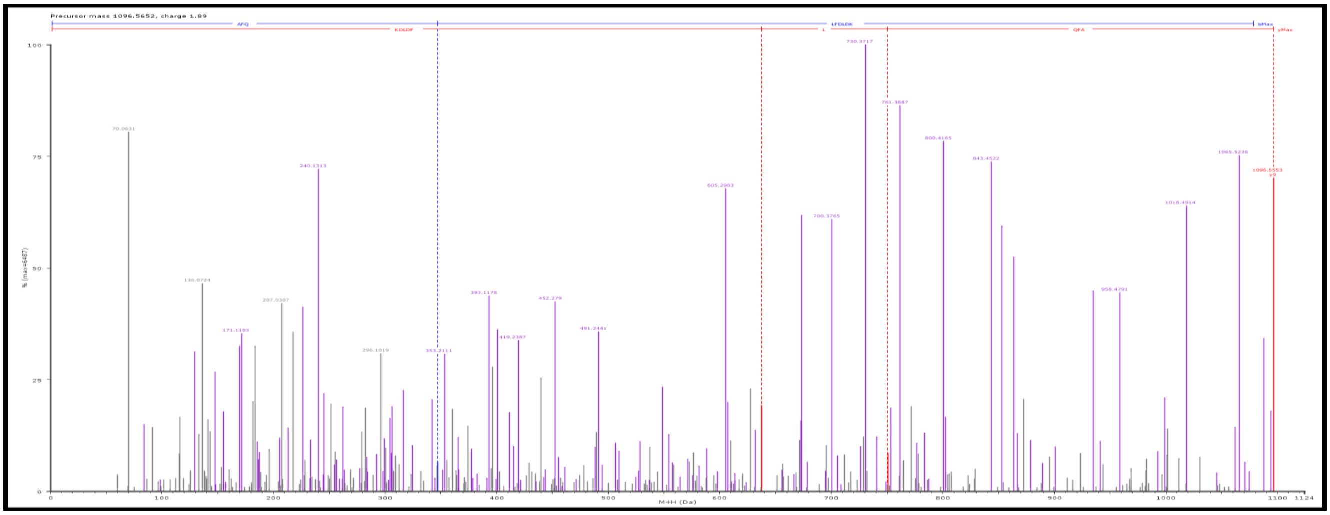

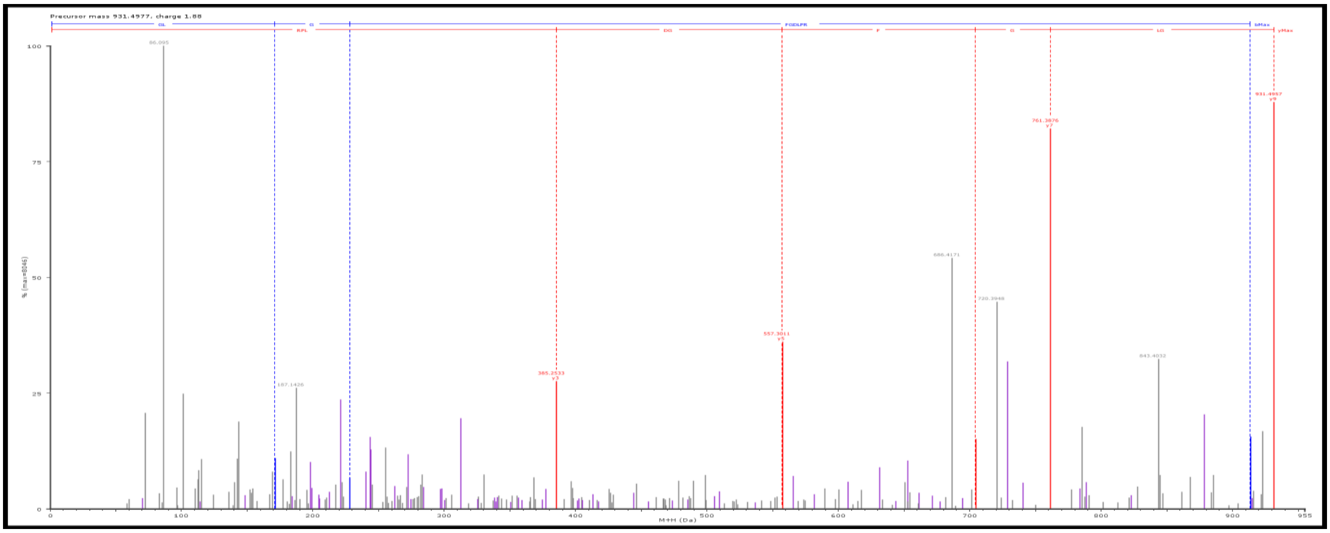

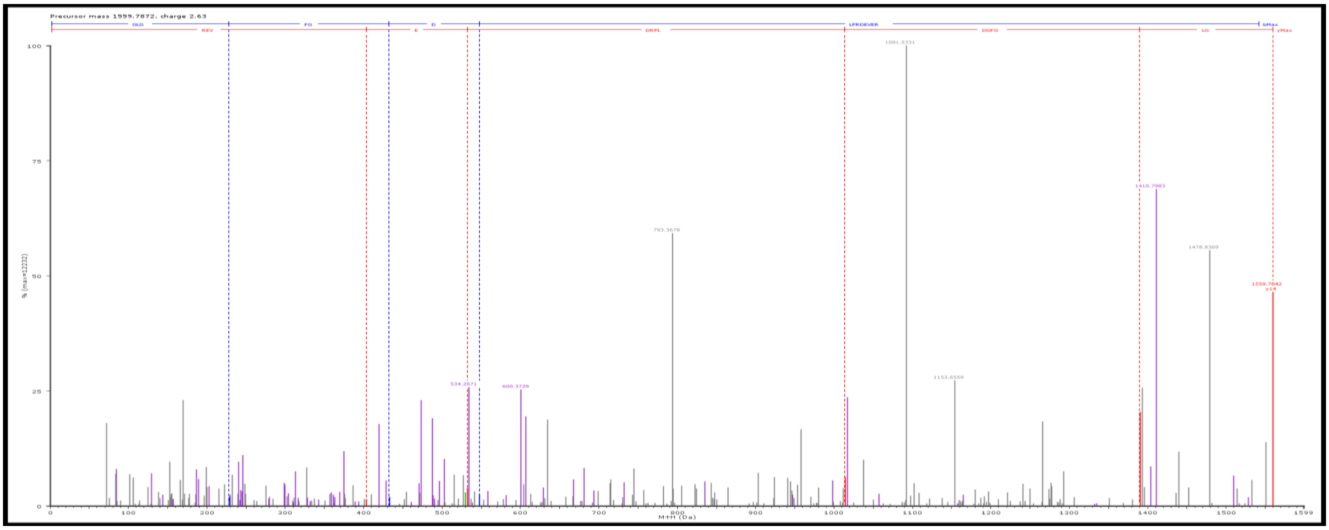

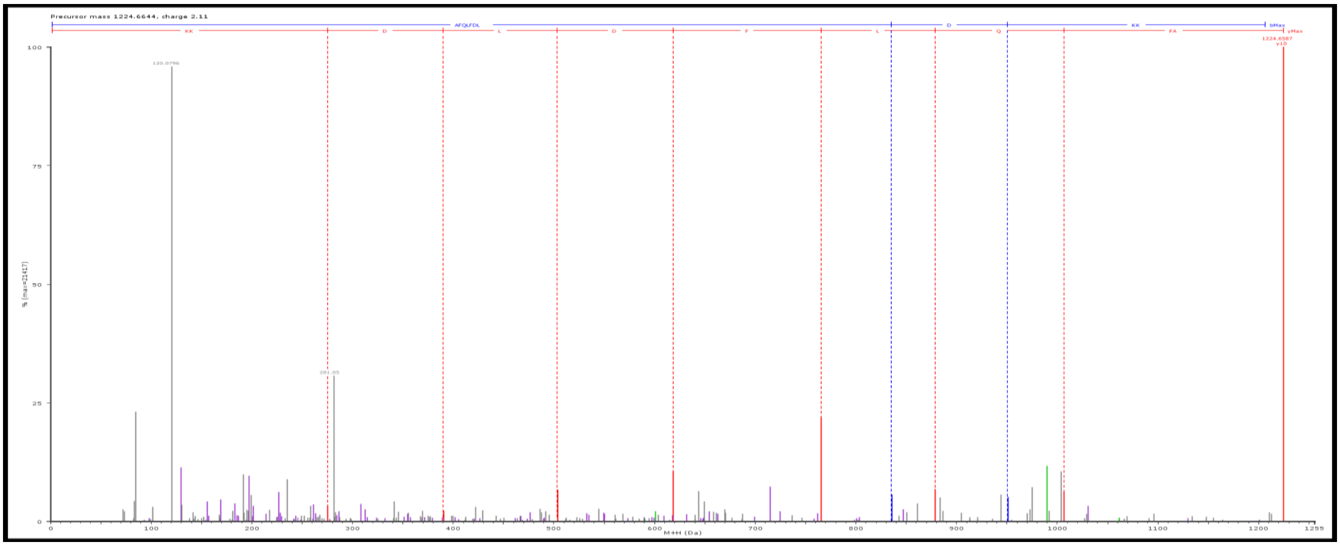
Precursor mass 1224.6644, charge 2.11
M+H (Da)

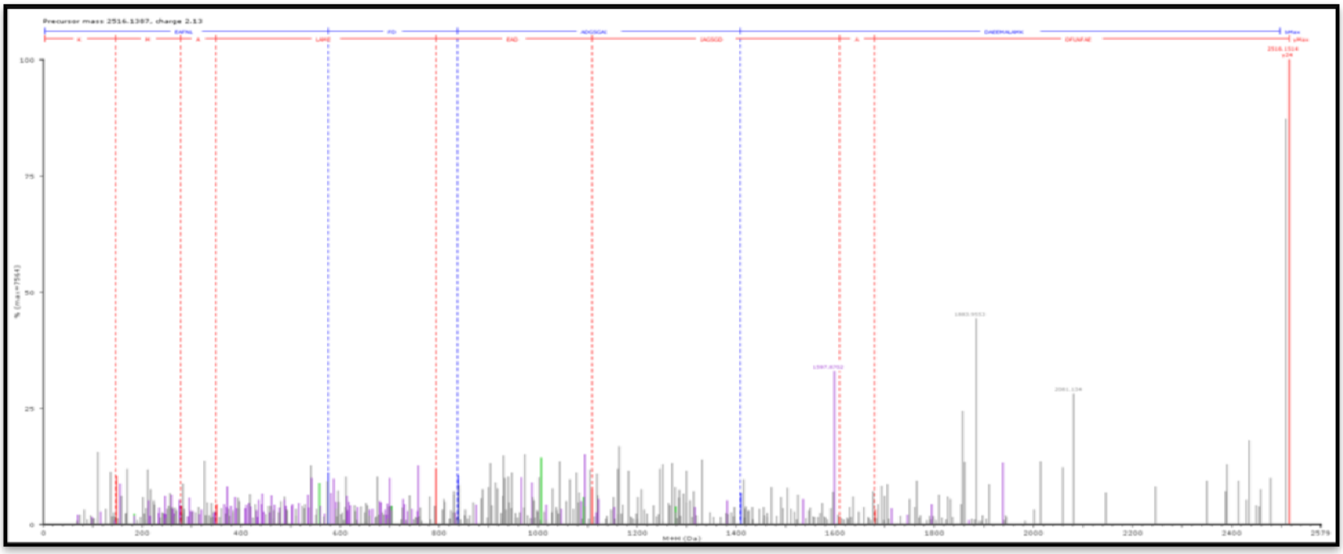
Precursor mass 2516.1387, charge 2.13
M+H (Da)

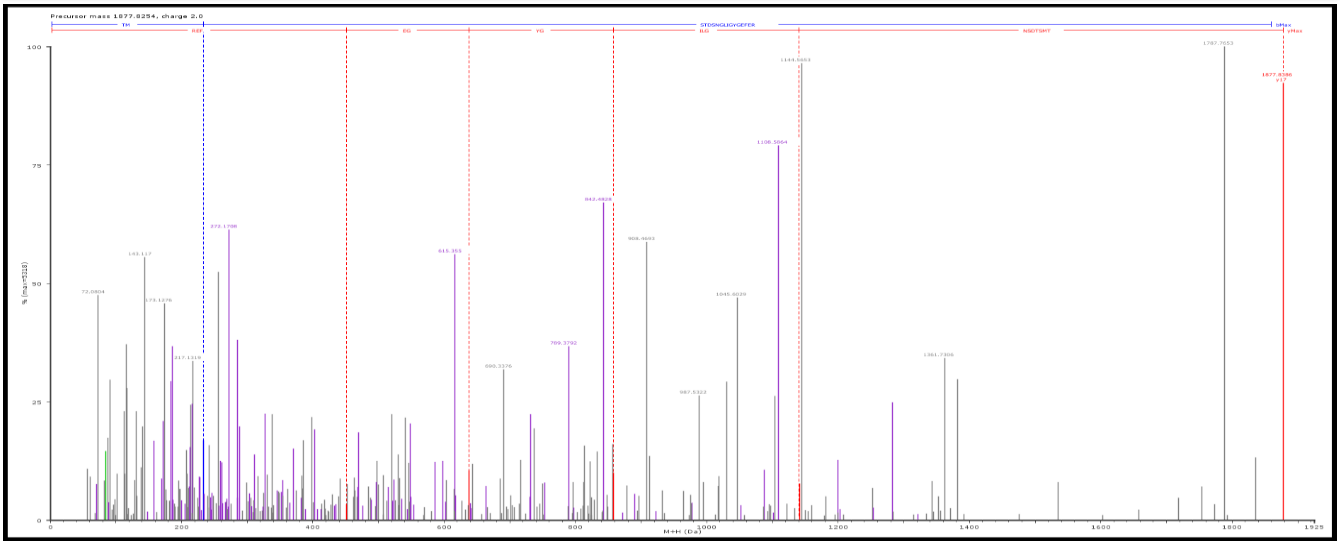
Precursor mass 1877.8254, charge 2.0
M+H (Da)

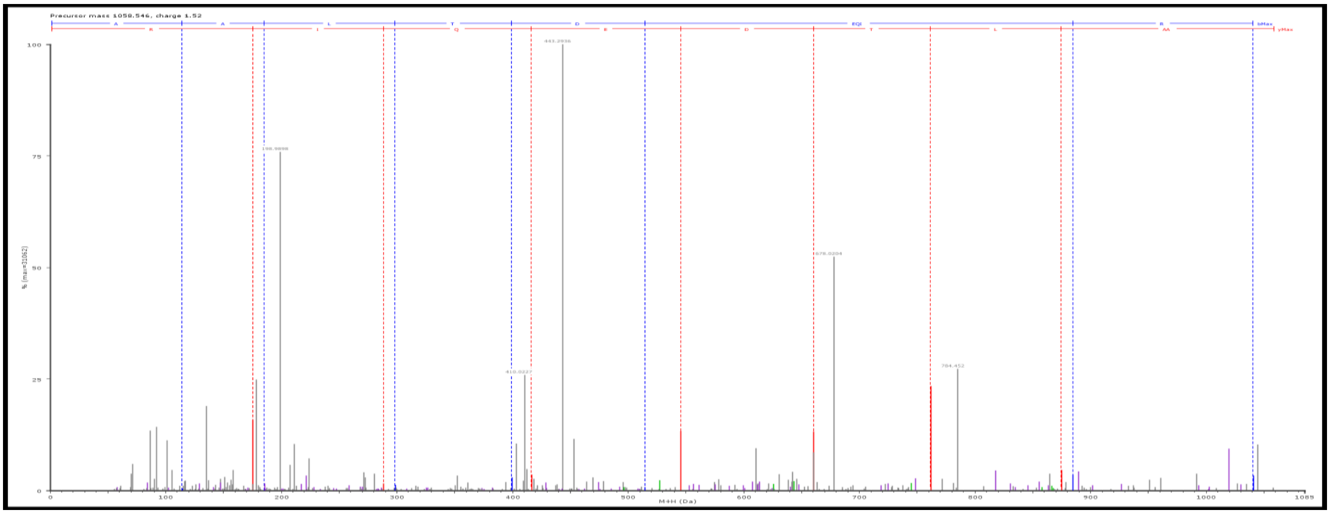

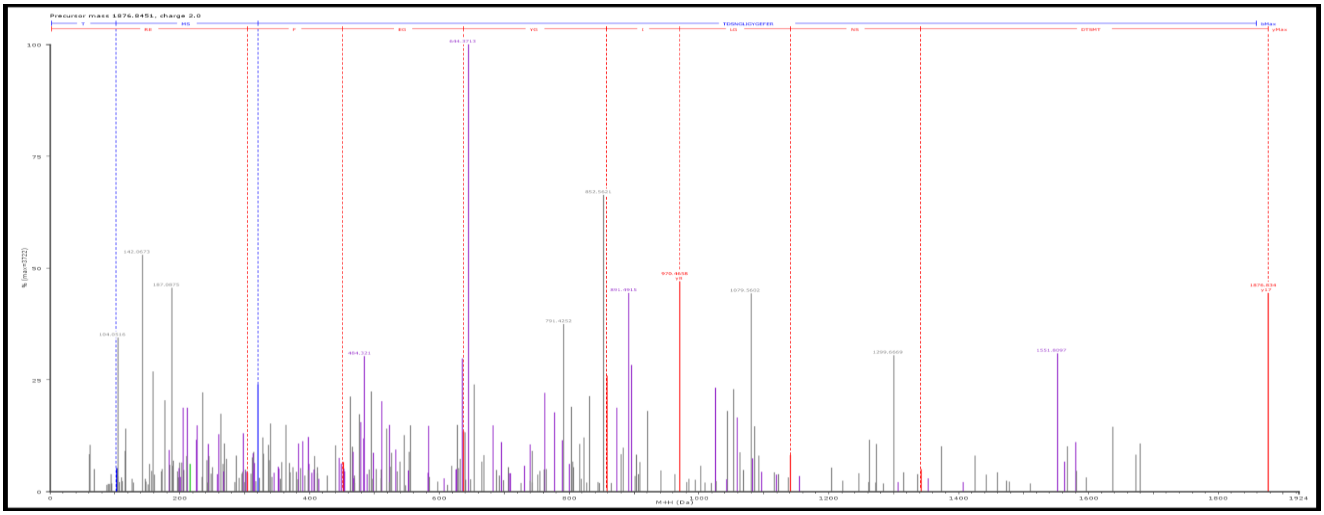

Protein entry	Protein description	Peptide sequence	Peptide score
Q4DYA8	Histone H2A Trypanosoma cruzi (strain CL Brener) Tc00.1047053511817.180 3 1	HDDDLGMLLK	8,772
		AGLIFPVGR	8,0815
		DVTLSR	7,7869

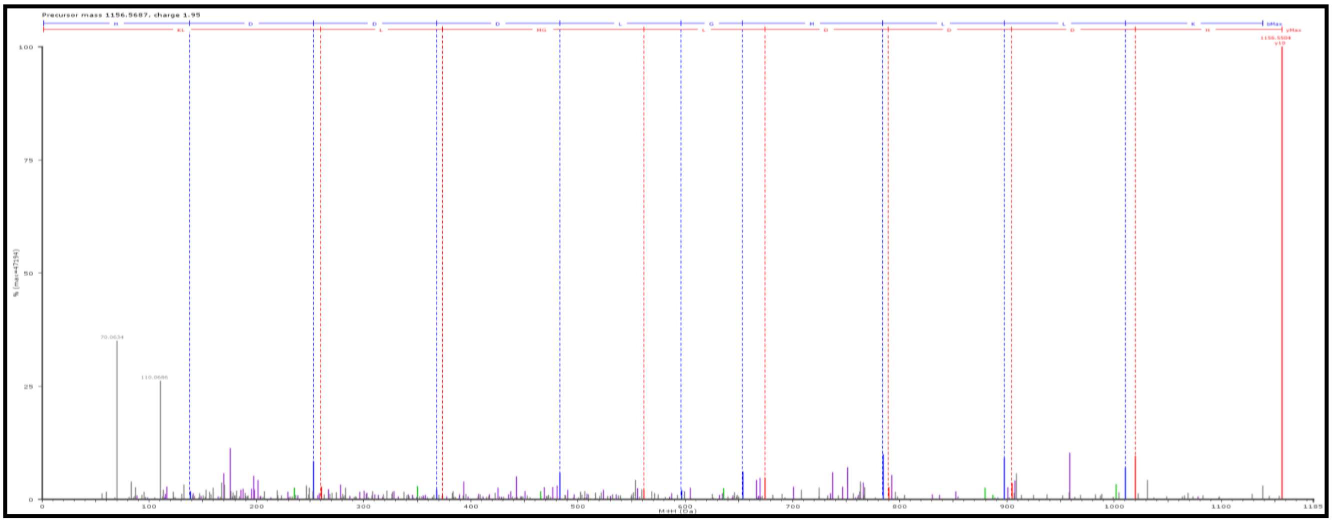

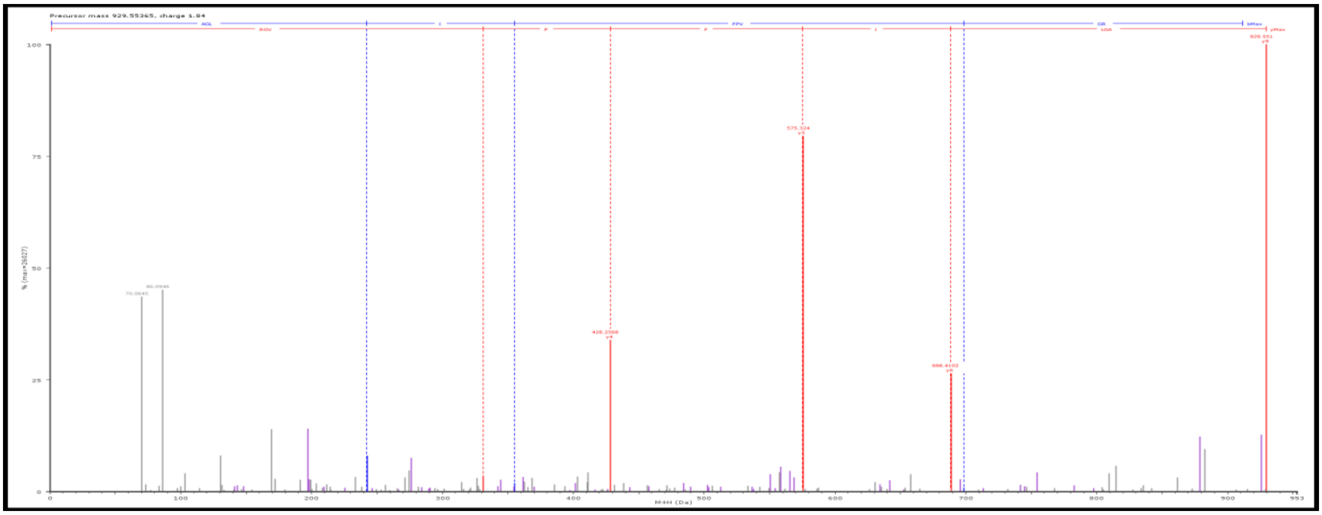

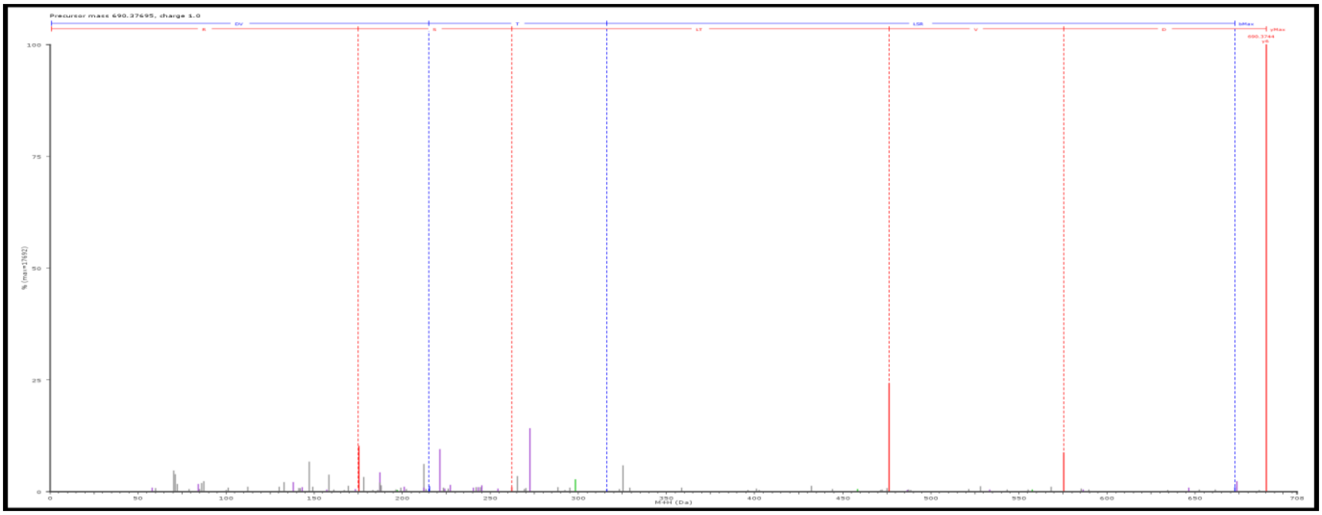

Protein entry	Protein description	Peptide sequence	Peptide score
Q4CTD7	Histone H2B Trypanosoma cruzi (strain CL Brener) Tc00.1047053511635.20 3 1	IVNSFVNDLFER	8,3939
		SINNHMSMSGR	8,5658
		TWNVYISR	8,3687
		LVLPADLAK	7,7043
		IVNSFVNDLFER	7,4619

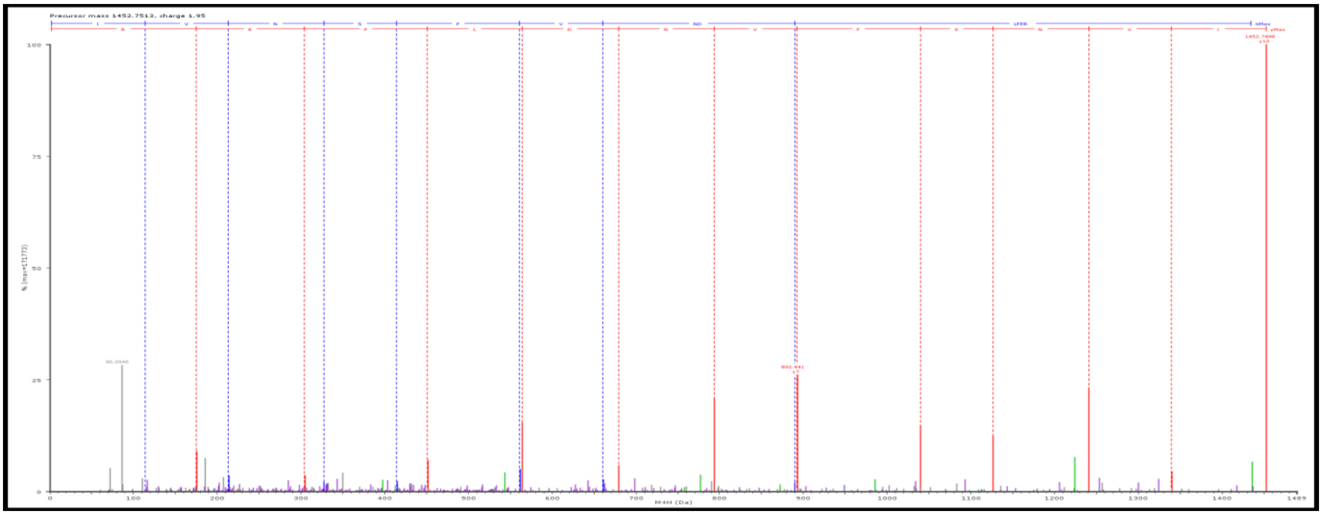

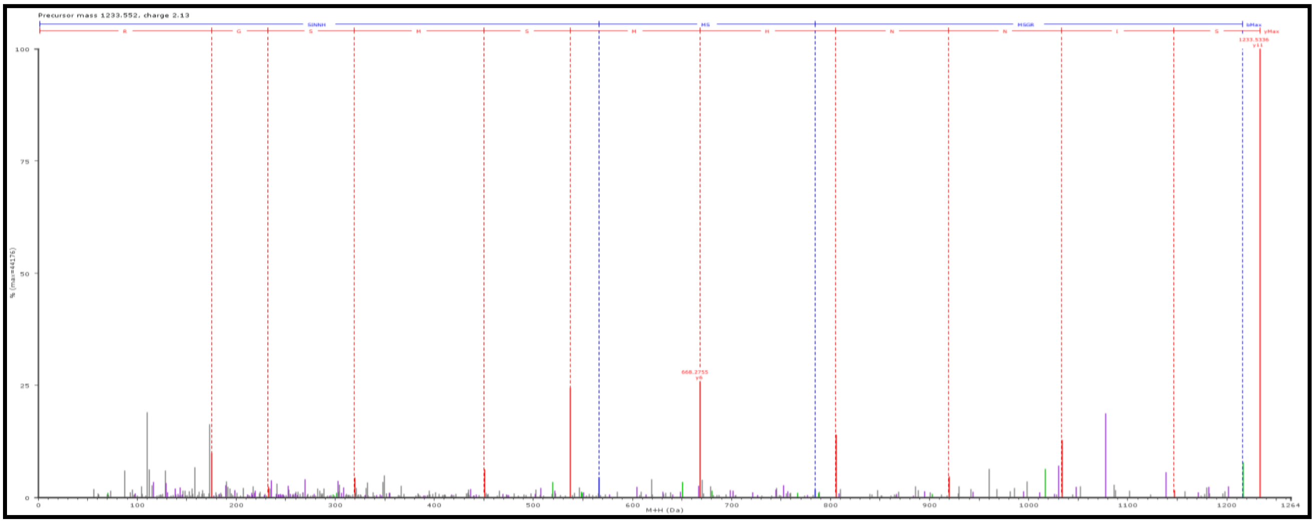
Precursor mass 1233.552, charge 2.13
M+H (Da)

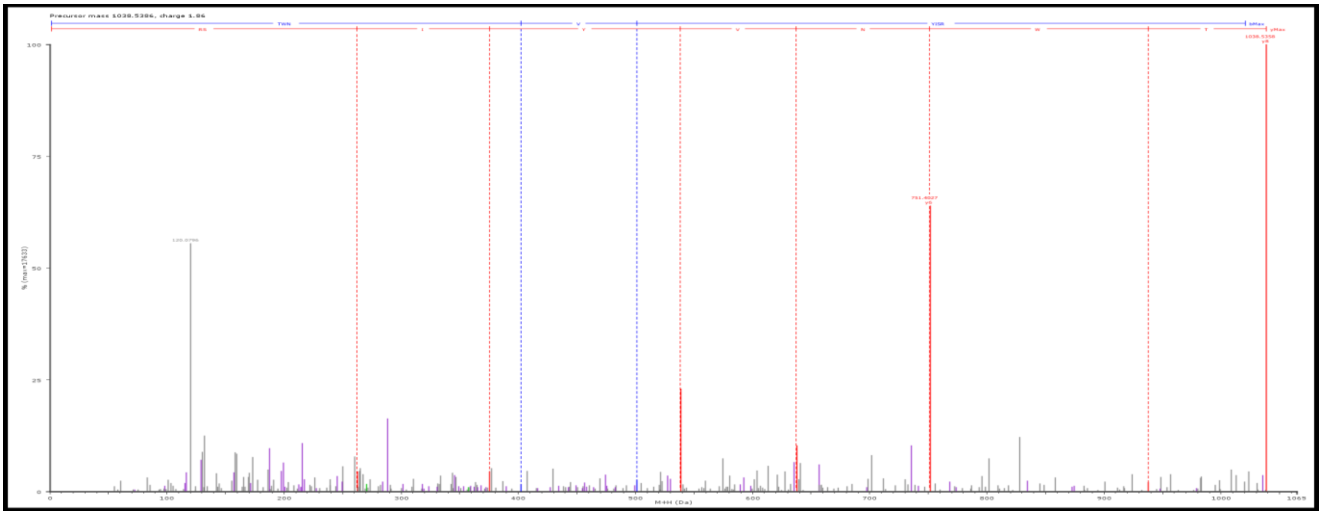
Precursor mass 1038.5386, charge 1.86
M+H (Da)

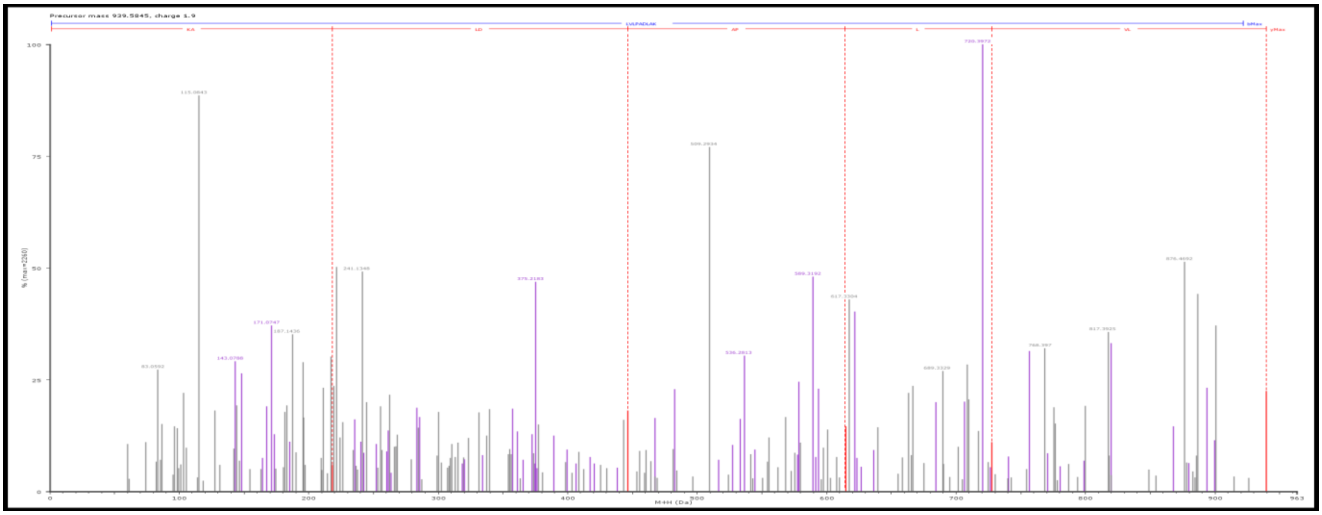
Precursor mass 939.5845, charge 1.9
M+H (Da)

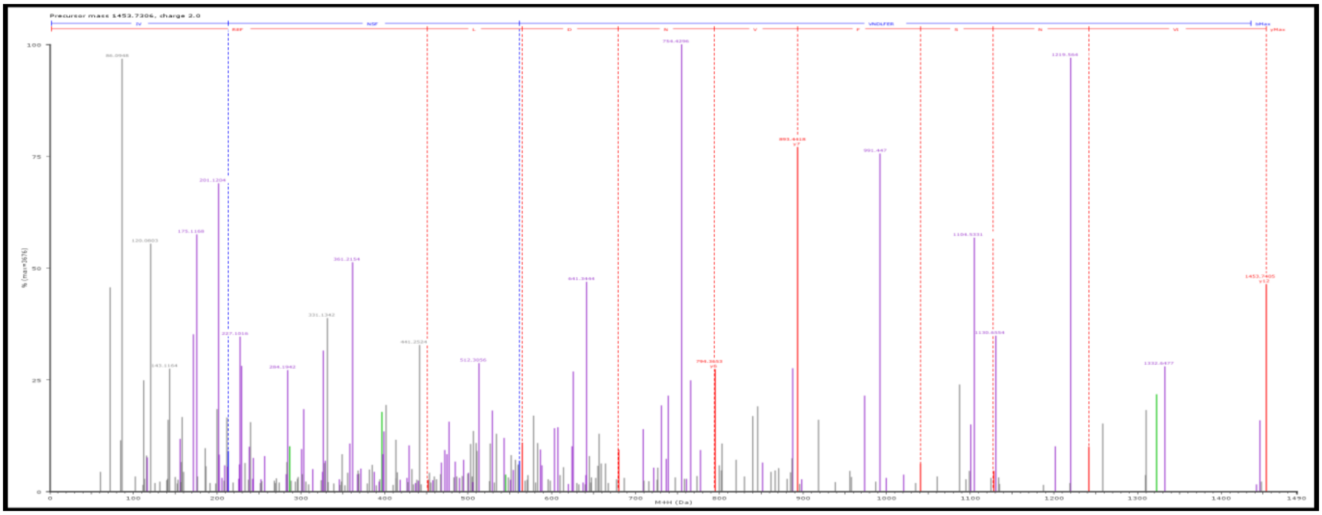

Protein entry	Protein description	Peptide sequence	Peptide score
Q4D0J9	Histone H3_ putative Trypanosoma cruzi (strain CL Brener) Tc00.1047053505931.40 4 1	FQSSAILAAQEATESYVVSLLADT NR	8,8546
		DIHLALCLR	7,235

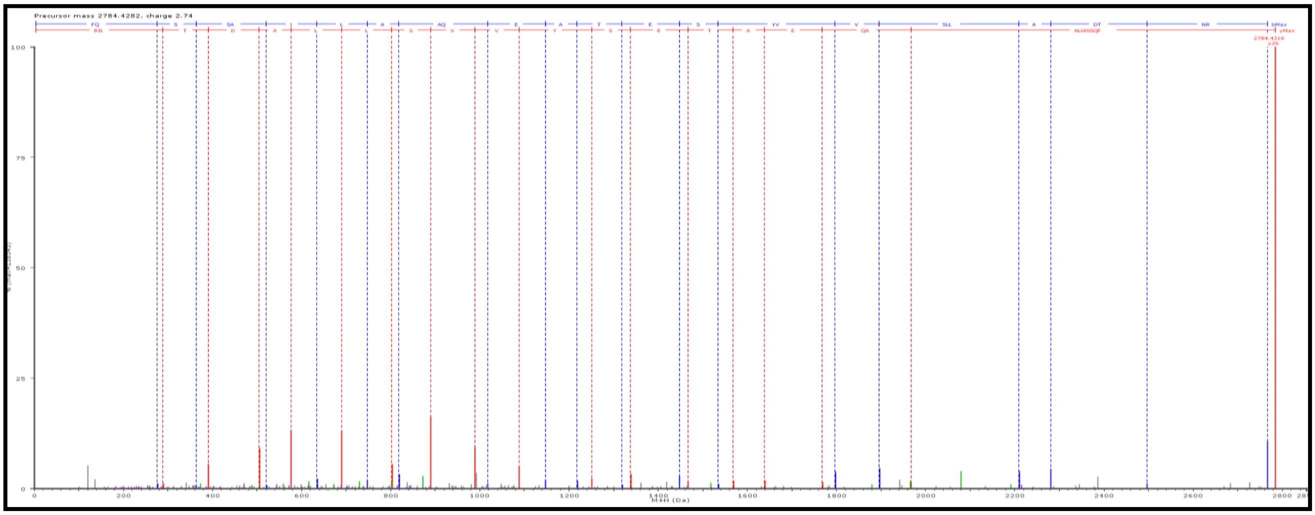

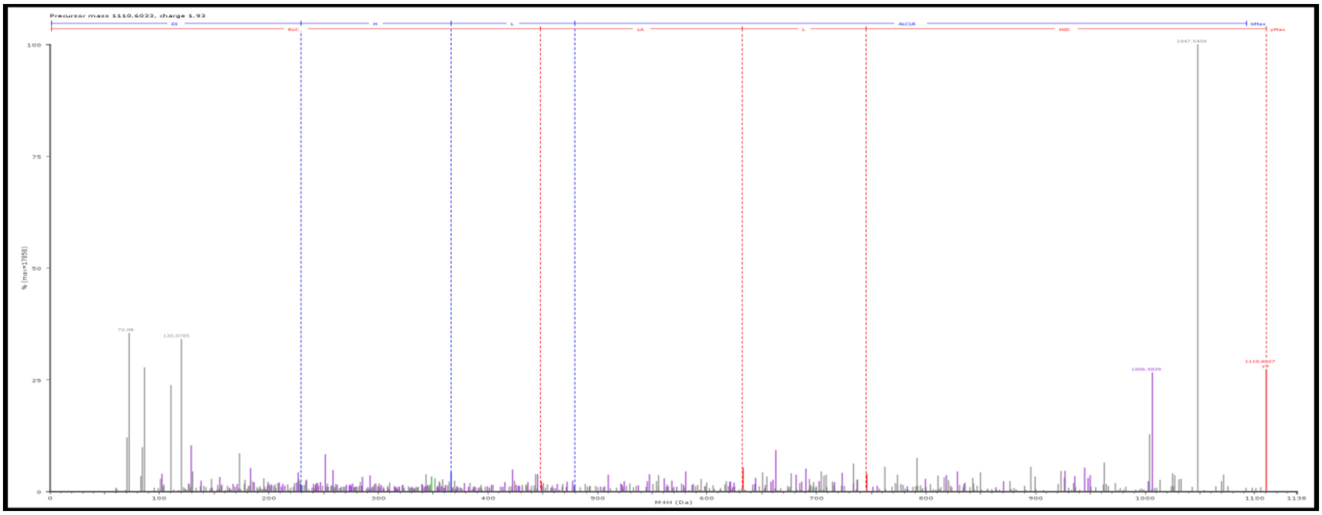

Protein entry	Protein description	Peptide sequence	Peptide score
Q4DEL1	Histone H4 Trypanosoma cruzi (strain CL Brener) Tc00.1047053507941.150 3 1	TVTAVDVVNALR	9,1406
		TVTAVDVVNALRK	7,6205
		SFVEGVVR	8,4695
		ISGVIYDEVR	8,7713
		KTVTAVDVVNALR	7,1371

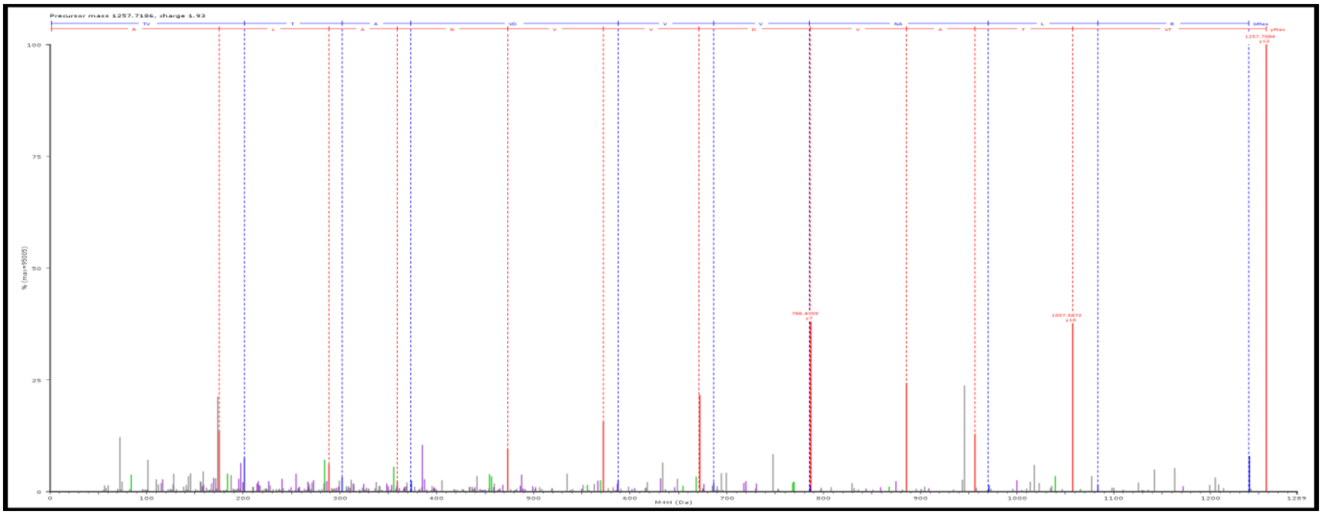

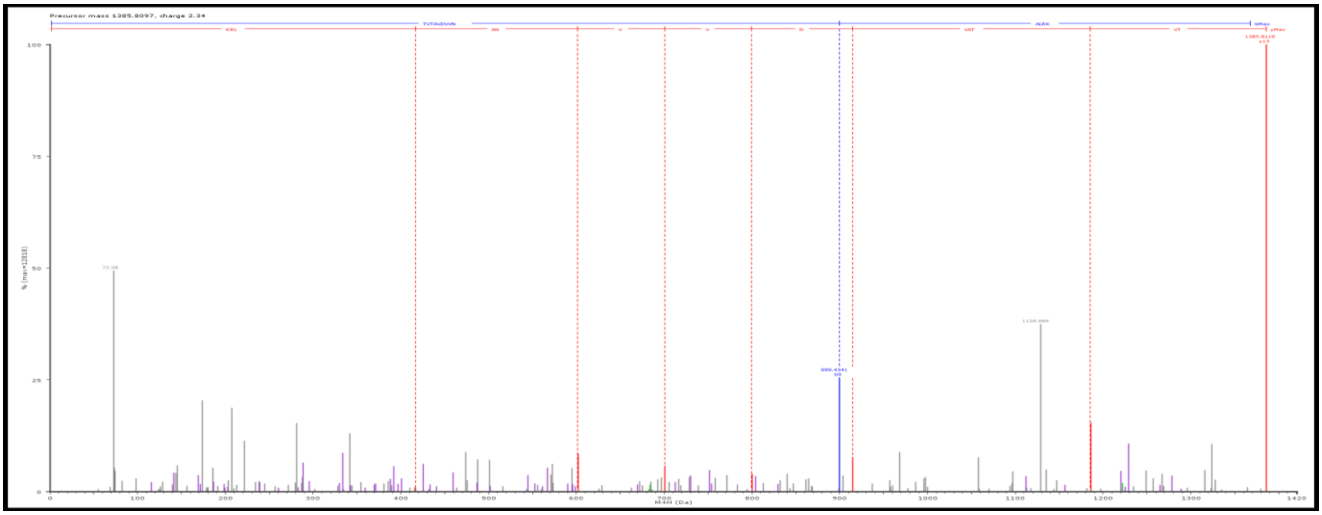

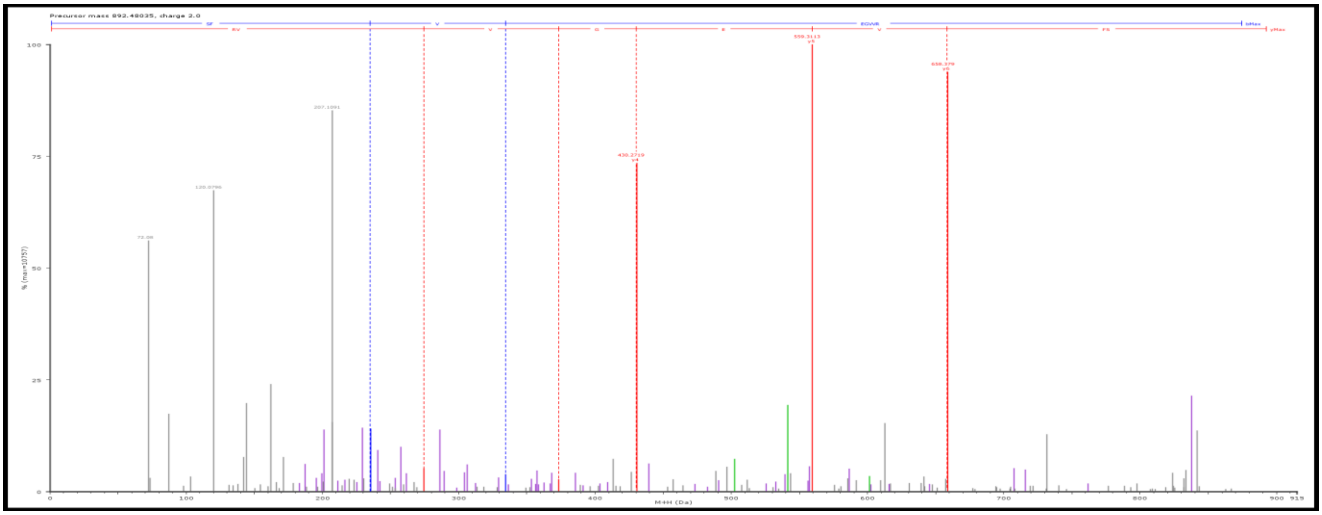

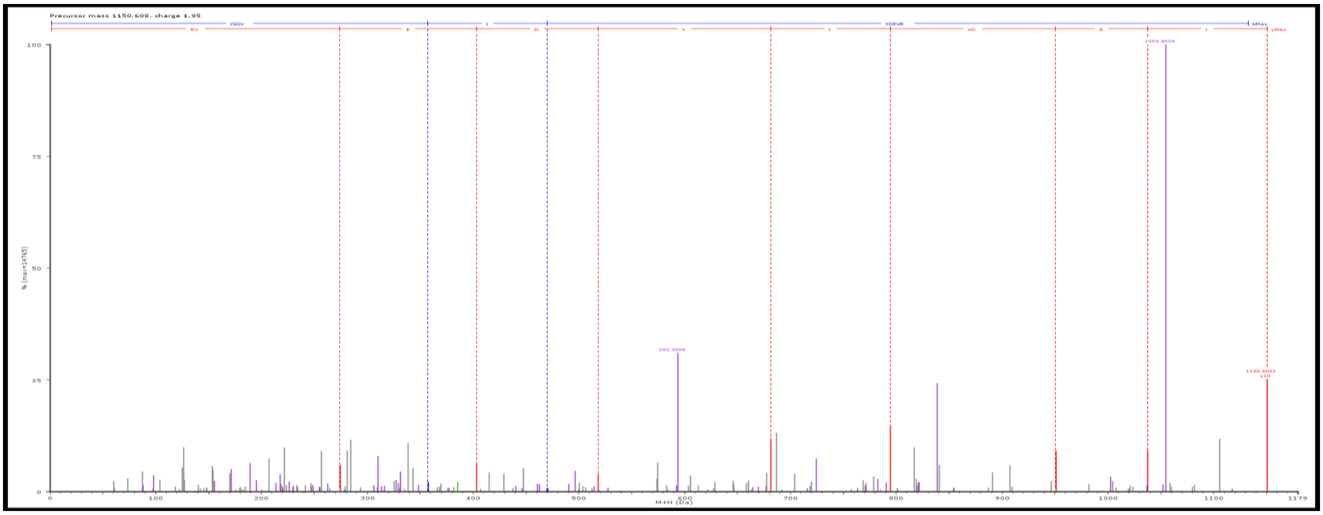

Precursor mass 1150.608, charge 1.95
ISGV
I
YDEVR
bMax
RV
E
D
Y
I
VG
S
I
yMax
1054.4624
593.3059
1150.6021
y10
% (max=1765)
M+H (Da)

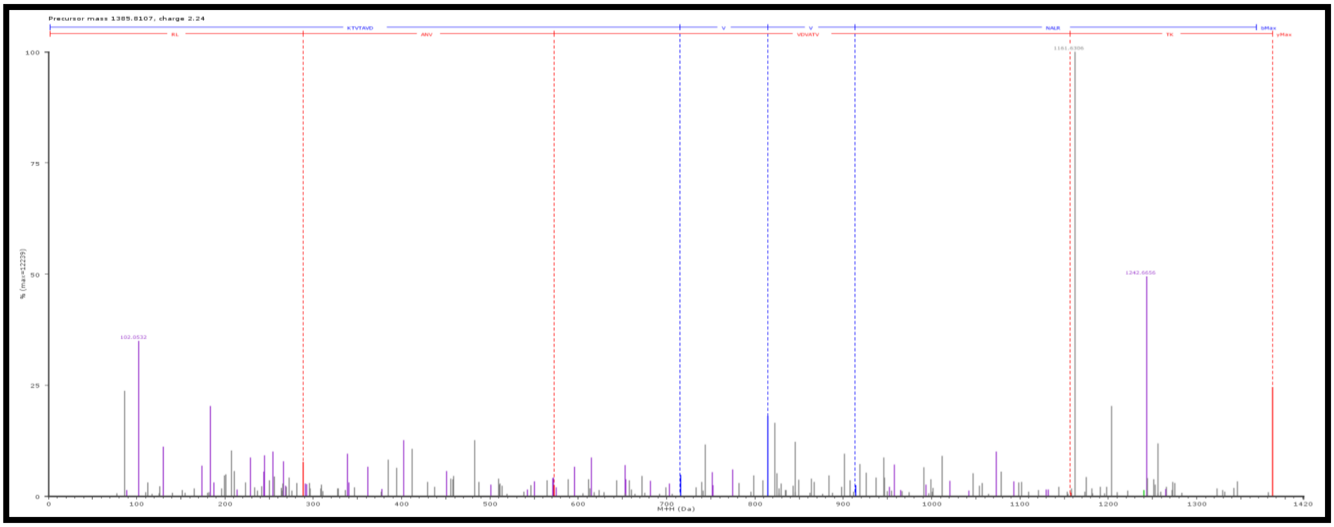

Precursor mass 1385.8107, charge 2.24
KTVTAVD
V
V
NALR
bMax
RL
ANV
VDVATV
TK
yMax
1161.6306
1242.6656
102.0532
M+H (Da)
